# Sino-European Transcontinental Basic and Clinical High-Tech Acupuncture Studies—Part 1: Auricular Acupuncture Increases Heart Rate Variability in Anesthetized Rats

**DOI:** 10.1155/2012/817378

**Published:** 2012-02-21

**Authors:** Xin-Yan Gao, Kun Liu, Bing Zhu, Gerhard Litscher

**Affiliations:** ^1^Department of Physiology, Institute of Acupuncture and Moxibustion, China Academy of Chinese Medical Sciences, Dongzhimen Nanxiaojie Street, Beijing 100700, China; ^2^TCM Research Center Graz and Research Unit of Biomedical Engineering in Anesthesia and Intensive Care Medicine, Medical University of Graz, Auenbruggerplatz 29, 8036 Graz, Austria

## Abstract

Evidence-based research concerning the effects of high-tech acupuncture on autonomic function was performed by two research teams from China and Austria. This study describes the first transcontinental teleacupuncture measurements in animals. Heart rate (HR) and heart rate variability (HRV) recordings in 10 male Sprague-Dawley anesthetized rats were performed under stable conditions in Beijing, China, and the data analysis was completed in Graz, Austria. The electrocardiograms (ECGs) were recorded by an HRV Medilog AR12 system during acupuncture of the ear and body (PC6 Neiguan, CV12 Zhongwan, ST36 Zusanli). The data were analyzed using specially adapted novel Austrian software. HR did not change significantly during any acupuncture stimulation in anesthetized rats (ear acupuncture, PC6, CV12, or ST36). Total HRV only changed significantly (*P* = 0.025) during auricular acupuncture (acupoint heart). The low-frequency/high-frequency ratio parameter decreased significantly (*P* = 0.03) during stimulation of ST36. This change was based on intensification of the related mechanism of blood pressure regulation that has been demonstrated in previous studies in humans. Modernization of acupuncture research performed as a collaboration between China and Austria has also been demonstrated.

## 1. Introduction

Acupuncture is being recognized as an effective treatment for various autonomic disorders; however, most of the mechanisms of this therapeutic method remain unclear. Evidence-based research and review studies on the effects of acupuncture on autonomic function have already been performed by the authors' research groups in the past [1–8]. The results obtained using high-tech methods are well documented and are important for general acceptance of this traditional Chinese medical treatment in the Eastern and Western world.

This study represents the first time that transcontinental teleacupuncture [3, 9] measurements have been performed in experimental animals. The main goal was to register and analyze the effects of different acupuncture stimulation on heart rate (HR) and heart rate variability (HRV) in anesthetized rats under stable conditions. The data were recorded for 10 rats in Beijing, China, and the data analysis was completed in Graz, Austria. A new HRV system partially developed in Austria was used to record the data in China, and the software that is normally used for human data analysis has been specifically adapted for this study in rats.

## 2. Materials and Methods

### 2.1. Animals

Experiments were conducted in accordance with *the Guide for Care and Use of Laboratory Animals* issued by the National Institutes of Health, and the procedures were approved by the Institutional Animal Care and Use Committee of the China Academy of Chinese Medical Sciences. Ten male Sprague-Dawley rats, weighing 300–350 g, were kept in an animal house maintained at 21 ± 2°C with a 12-hour light-dark cycle and were given free access to food and water. The animals were initially anesthetized with an intraperitoneal injection of 10% urethane (1.0 g/kg, Sigma-Aldrich, St. Louis, USA). The left common carotid artery was cannulated with a polyethylene catheter filled with physiological saline containing heparin (200 IU/mL, Sigma-Aldrich, St. Louis, USA) to record arterial pressure (AP) via a blood pressure transducer (DA100, Biopac Systems Inc., Aero Camino Goleta, USA) and amplifier (MP150, Biopac Systems Inc., Aero Camino Goleta, USA). This signal was registered on Micro1401 and Spike2 (CED, Cambridge Electronic Design Limited, Cambridge, UK) data acquisition unit and software. The depth of anesthesia was monitored by changes in AP, and additional anesthetic (urethane 0.3 g/kg) was given if the animal showed large fluctuations in baseline AP or a withdrawal response to a pinch of the paw. After tracheal cannulation, the animals breathed spontaneously, and their core temperature was maintained at 37.0 ± 0.5°C by a feedback-controlled electric blanket (FHC Inc., Bowdoin, USA). The animals were sacrificed after the investigation by an overdose of anesthetics.

### 2.2. Electrocardiographic Monitoring

The electrocardiograms (ECGs) were recorded by an HRV Medilog AR12 (Huntleigh Healthcare, Cardiff, UK, and Leupamed GmbH, Graz, Austria) system. The data were analyzed using specially adapted software (Huntleigh Healthcare). The system was designed for a monitoring period of more than 24 hours, and the sampling rate of the recorder is 4096 Hz, allowing R-waves to be detected extremely accurately. All raw data from the rat experiments were stored digitally on a 32 MB compact flash memory card. After removing the card from the portable system in the lab in Beijing, the data were read by an appropriate card reader connected to a standard computer and sent to the lab in Graz. The dimensions of the HRV recorder are 70 × 100 × 22 millimeters, and the weight is approximately 95 grams with batteries [10].

**Figure 1:**
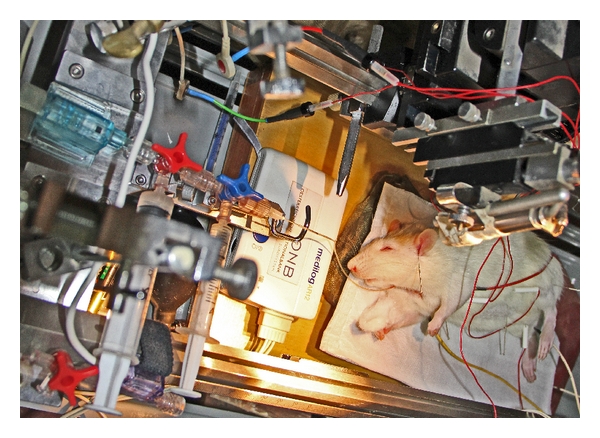
Transcontinental animal experiment using rats in Beijing at the Institute of Acupuncture and Moxibustion at China Academy of Chinese Medical Sciences. HRV equipment from the TCM Research Center Graz was used, and the data analysis was performed at the Medical University of Graz in Austria.

To collect ECG data in rats, which have hairy skin, three electrode plates designed for use in humans were adapted by connecting them to three needle electrodes that were then placed separately in subcutaneous muscles (Figure 1).

HRV is measured as a percent change in sequential chamber complexes called RR-intervals in the ECG. It can be quantified in the time domain and in the frequency range by analyzing the ECG power spectra [1, 3, 4, 8, 10, 11]. The HRV parameters are recommended by the task force of the European Society of Cardiology and the North American Society of Pacing and Electrophysiology [11]. Using new software (Huntleigh Healthcare, Cardiff, UK), the HRV is analyzed and displayed in a novel way to evaluate the function of the autonomic nervous system [10]. The mean HR, total HRV, and LF (low frequency)/HF (high frequency) ratio of the HRV were evaluated [11].

### 2.3. Acupuncture Stimulation and Procedure

The auricular point “Heart” and body points were selected, including PC6 (Neiguan) as the homotopic point that has the same segmental innervation as the heart and ST36 (Zusanli), as the heterotopic point that has different segmental innervation compared to the heart. Both points regulate cardiovascular functions. CV12 (Zhongwan) was selected as another heterotopic body point and is reported to regulate gastrointestinal function. All points were identified by anatomical marks based on descriptions in textbooks and previous reports [2, 12–14]. Briefly, PC6 is located proximal to the accessory carpal pad of the forelimb between the flexor carpi radialis and palmaris longus ligaments. CV12 is located in the medioventral line, 3 mm above the umbilicus. ST36 is located on the anterolateral side of the hindlimb near the anterior crest of the tibia below the knee under the tibialis anterior muscle. The auricular point “Heart” is located at the inferior concha [2, 12, 15].

**Figure 2:**
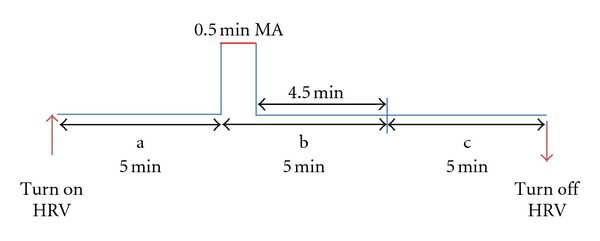
Experimental protocol for manual acupuncture (MA) at the auricular point Heart and body points.

For manual acupuncture stimulation, needles (length: 13 mm, diameter: 0.2 mm; Hwato, China) were inserted perpendicularly to the skin to a depth of 2 mm at the auricular point (Heart) and 4-5 mm at somatic points. When the fluctuations in arterial blood pressure were less than 5%, acupuncture stimulation was applied with neutral supplementation and draining manipulation by twisting the needle for 30 sec. The time course of each stimulation is shown in Figure 2. The order of point stimulation was randomized, and the time between the investigations of the different acupoints was at least 10 minutes.

The measurement profile and measurement times (a–c) are shown schematically in Figure 2. Three measurement periods were compared: one before stimulation (a) one immediately after 30 sec of acupuncture stimulation (b) and one as a second control (c).

### 2.4. Statistical Analysis

The data were analyzed using one-way repeated measures analysis of variance (ANOVA) (SigmaPlot 11.0, Systat Software Inc., Chicago, USA). Post hoc analysis was performed using Tukey and Holm-Sidak tests. The level of significance was defined as *P* < 0.05.

## 3. Results

**Figure 3:**
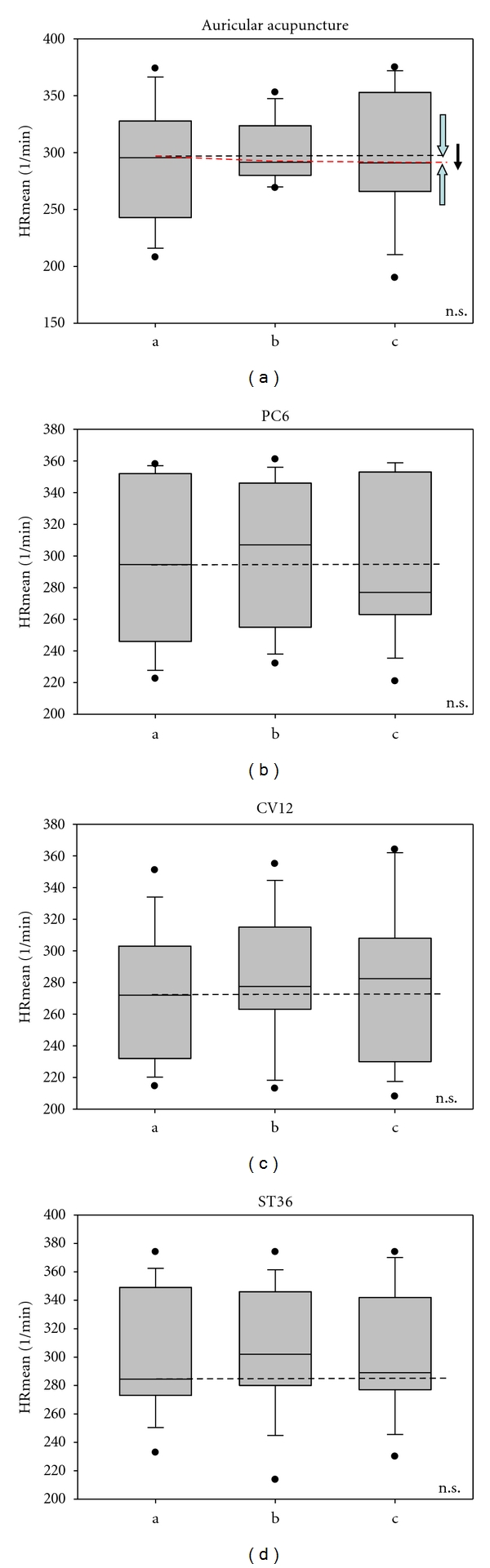
Box plots displaying the mean heart rate (HRmean) of the 10 rats. There are no significant differences. The ends of the boxes define the 25th and 75th percentiles with a line at the median and error bars defining the 10th and 90th percentiles. The different measurement phases (a–c; compare with Figure 2) and acupuncture points (auricular acupuncture: Heart point, PC6, CV12, and ST36) are indicated.

**Figure 4:**
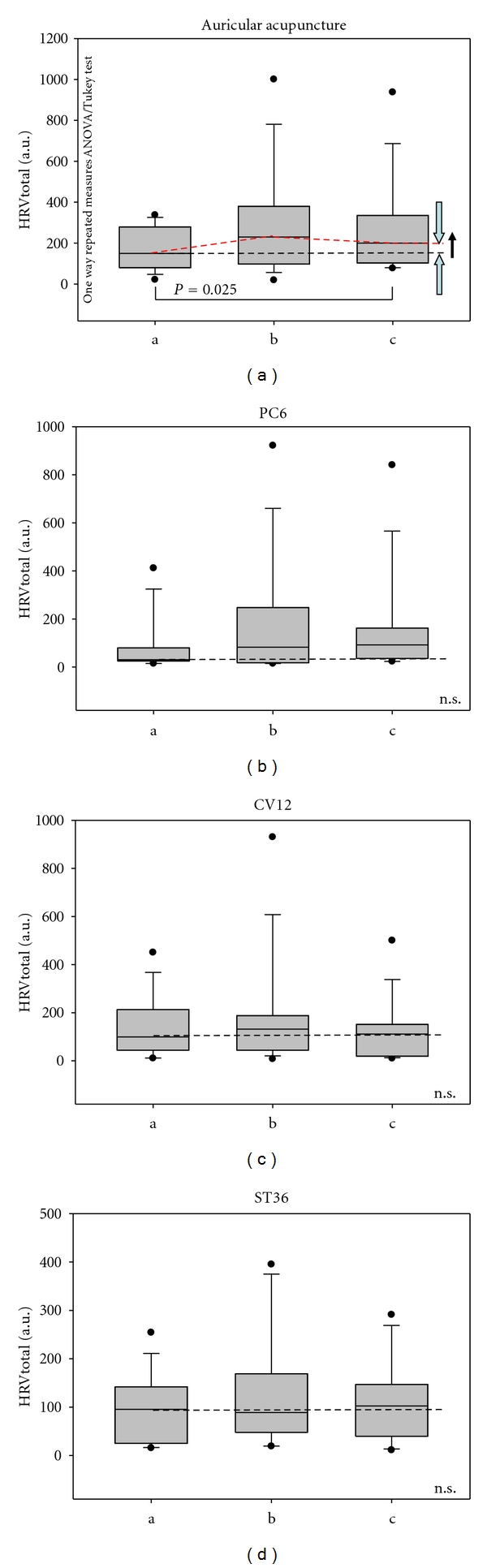
Box plots displaying total heart rate variability (HRV total) for the 10 rats. Note the significant increase in HRV total after auricular acupuncture. For further explanation, compare with Figure 3.

Figures 3 and 4 show the mean HR and HRV total (total heart rate variability) from the ECG recordings from the 10 rats during the three measurement phases (a, b, and c) as well as before, during, and after stimulation at the “heart” ear acupoint. The results from the stimulation of the body points are also shown (PC6, CV12, and ST36). There was no significant change in HR during the stimulation sessions (Figure 3).

HRV total increased significantly (*P* = 0.025) only after manual ear acupuncture at the point Heart (Figure 4).

**Figure 5:**
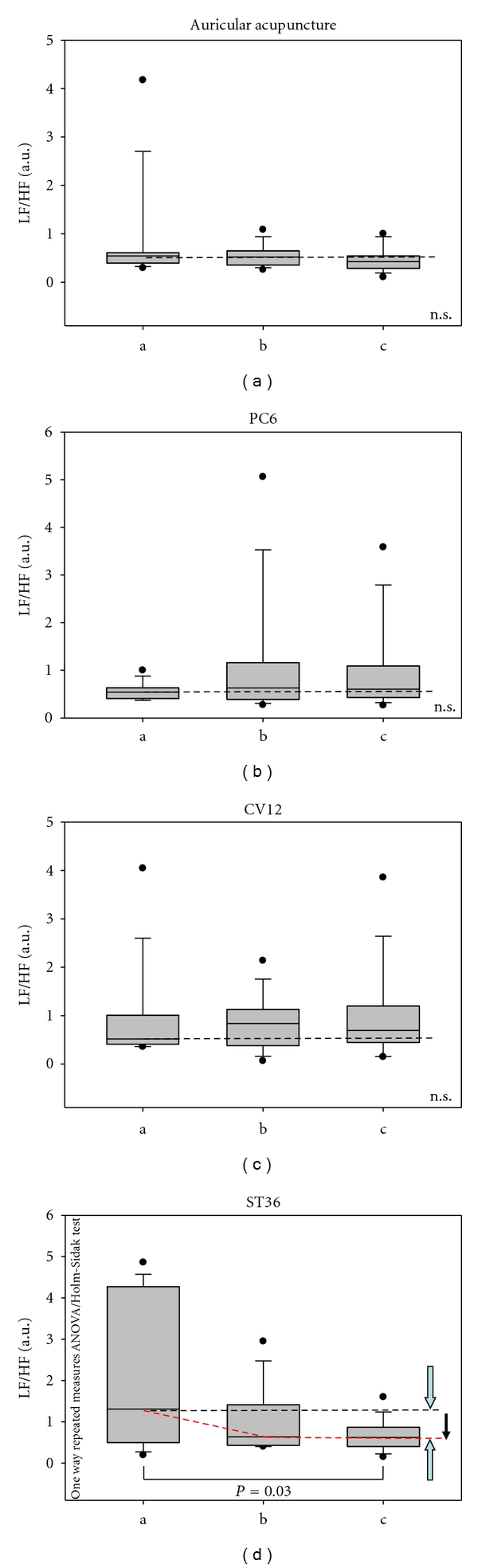
LF (low frequency)/HF (high frequency) ratio. Note that the median of the LF/HF parameter decreases after acupuncture in the ten rats. For further explanation, see Figures 4 and 5.

Furthermore, continuous HR-HRV monitoring showed substantial and significant (*P* < 0.03) decreases in the LF/HF ratio after acupuncture stimulation at ST36 (Figure 5).

## 4. Discussion

In 1858, the first transatlantic telecable between Ireland and Newfoundland was installed. It was not successful and worked only for a short time period; however, this was one of the first communication connections between different countries. The first transatlantic connection was then realized in 1866. This connection was the beginning of transmitting data between continents and was the first step towards today's medical information technology.

Today, teleacupuncture between Europe and Asia is no longer a vision of the future. It has already become a reality, and we have described this new approach in previous studies [3, 9]. Up to now, we have performed these measurements in humans. This study is the first using teleacupuncture in an animal experiment.

Measurement of beat-to-beat HRV has been shown to provide a good estimation of autonomic control [1, 8, 11]. Power spectral analysis of HRV is a well-documented method in humans [1, 3, 4, 8–11]; however, there are only a few studies in rats [2, 12–20]. The HR of rats is much higher than that of humans, and therefore, the RR-intervals in rats are much smaller than those in humans or dogs. Thus, we developed and adapted the software to allow for adequate resolution of the ECG signals. The novel “fire of life” analysis program [10] from TOM Medical Development (GmbH, Graz, Austria) was used.

Standardization of the evaluated parameters in rats has not yet been performed, so results from different studies cannot be compared. For example, different methods are used to determine the LF/HF ratio, as different studies use different frequency bands. Hashimoto et al. [16] defined the LF with 0.04–1 Hz and the HF with 1.0–3.0 Hz. In contrast, Gao et al. [17] defined the LF band with 0.04–0.15 Hz and the HF band with 0.15–0.40 Hz. Kuwahara et al. [18] also used the LF/HF ratio but employed yet another set of ranges: LF (0.04–1.0 Hz) and HF (1.0–3.0 Hz). The study conducted by Kuwahara et al. is from the same group in Japan as the study conducted by Hashimoto et al. [16]. In the studies by Shen et al. [19] and Li et al. [14], no numerical values for the LF and HF edge frequencies can be found. We defined our ranges according to previous HRV studies in rats [20], with LF < 0.5 Hz and HF ≥ 0.5 Hz up to the Nyquist frequency as determined by the mean RR-interval of the tachogram [20]. From each unconscious rat, ECG signals were recorded continuously during steady state conditions over a period of 15 minutes (see Figure 2). In addition to HRV total, Kuwahara et al. [18] stated that the LF/HF ratio seems to be a convenient index of parasympathetic and sympathetic interactions in the rat. In our investigation, this parameter decreased significantly after stimulating the acupoint, ST36.

One major finding of this study was a significant increase in HRV total after auricular acupuncture at the ear point Heart. This increase is very interesting as HR did not increase at the same time; on the contrary, HR decreased slightly (insignificantly) during and after auricular acupuncture (compare Figure 3). In agreement with these results, biomedical studies on 14 human subjects in Graz have indicated that the ear acupuncture point heart is a very important point in the regulation of the cardiocirculatory mechanism [4]. The results from the Graz study showed a significant decrease in HR and a significant increase in HRV total after manual ear acupressure at this ear acupuncture point, “Heart” [4].

Basic animal research concerning how acupuncture and acupuncture-like stimulations affect cerebral autonomic function has been performed by Gao et al. in two important previous studies [2, 12] on animal models. One study [2] aimed to examine the effects of acupuncture stimulation at different auricular areas on cardiovascular and gastric responses. Similar to this experiment, stimulation with manual acupuncture was performed in anesthetized Sprague-Dawley rats. They found that the largest depressor response was evoked from an area that corresponds to the “heart” stimulation point in humans that was also used in our study. Similar patterns of cardiovascular and gastric responses could be evoked by stimulating different areas of the auricle [2]. This does not support the theory of a highly specific functional map on the ear; rather, there is a similar pattern of autonomic changes in response to auricular acupuncture with variable intensity depending on the area of stimulation [2]. In light of these previous results, we used active manual stimulation methods applied at the same acupoint and did not perform acupuncture or acupuncture-like stimulation at a control point close to the stimulation area [2, 4].

The second important previous study from Gao et al. was published recently in 2011 in *Brain Research *[12] and demonstrated that auricular acupuncture induces cardiovascular inhibition, increases the response of cardiac-related neurons in the nucleus tractus solitarius, and evokes cardiovascular inhibition via the baroreceptor reflex. In that study, acupuncture-like stimulation was repeated in 58 male Sprague-Dawley rats in the area of the auricular point, “heart”. The authors clearly showed that acupuncture at this point regulates cardiovascular function by activating the cardiac-related and depressor neurons in the nucleus tractus solitarius in a manner similar to the baroreceptor reflex [12].

There are only a few experimental studies concerning acupuncture-like stimulation of the ear at the “heart” acupoint in humans [4, 21, 22]. In addition to the aforementioned Graz study [4], one study [21] demonstrated a marked hypotensive effect associated with stimulation of the “heart” point. The results of another investigation [22] indicated that auricular acupuncture plus needle-embedding at the “heart” acupoint could improve left cardiac function in patients with heart failure complicated by dilated cardiomyopathy and that the function of an acupoint is distinctly different from that of a nonacupoint.

The RR-interval is controlled by the system that regulates blood pressure, which is in turn influenced by the hypothalamus and, particularly, the vagal cardiovascular center in the lower brainstem [4, 11]. Some of the frequency bands in the ECG spectrum of the HRV can be interpreted as markers of physiological relevance. Several of the associated mechanisms are involved in regulating temperature (found in the very low-frequency band), blood pressure, and respiration [4, 11]. The following influences can be distinguished for different ranges of HRV in humans: (a) respiratory sinus arrhythmia (approximately 0.15–0.5 Hz), including central nervous system respiratory impulses and interactions with pulmonary afferents; (b) the so-called “10-s-rhythm” (approx. 0.05–0.15 Hz), which describes the natural rhythm of active cardiovascular neurons in the lower brainstem (the circulatory center and its modulation by feedback with natural vasomotor rhythms via baroreceptor feedback); and (c) longer wave HRV-rhythms (approx. <0.05 Hz), such as effects from the renin-angiotensin system and temperature regulation as well as metabolic processes [4, 11]. Although there are not many studies on ECG power spectral analysis in rats, similar frequency ranges can be determined as described previously [16–20].

In this study, HR decreased insignificantly at the same time that HRV total increased significantly during acupuncture of the ear (Figures 3 and 4). Manual ear acupuncture had a greater effect on HRV than acupuncture at points on the body (PC6, CV12, or ST36). The LF/HF ratio decreased significantly during acupuncture stimulation of ST36. This decrease could be mainly attributed to intensification of the related mechanism of blood pressure regulation (10-s-rhythm) as described in previous investigations in humans [4].

There are some limitations of this pilot study. The number of rats was small (*n* = 10), and there was no control group with a control nonacupuncture point. As already mentioned in a previous study by our two teams [4] and in the discussion, previous results from a study by Gao et al. [2] showed that it is difficult to identify a placebo point on the ear for such investigations. Our study design also does not allow conclusions concerning the underlying mechanism. This could be a topic for future investigations.

Progress can be made in high-tech acupuncture research by using modern biomedical techniques like teleacupuncture and analysis techniques like HRV “fire of life analysis” in animal experiments. Furthermore, modernization of acupuncture research performed as a collaborative effort between the Institute of Acupuncture and Moxibustion at the China Academy of Chinese Medical Sciences in Beijing and the TCM Research Center at the Medical University of Graz in Austria has been demonstrated in this study.

## 5. Conclusions

The following conclusions can be drawn from the results of this transcontinental experimental animal teleacupuncture study.

- Heart rate does not change significantly during acupuncture stimulation of the ear, PC6, CV12, or ST36 in anesthetized rats.
- Total HRV changes significantly during auricular acupuncture (acupoint heart), but not during stimulation of the other acupuncture points (PC6, CV12 or ST36). HRV total increases during auricular acupuncture, which improves the neurovegetative condition. This is interesting as the decrease in HR was not significant during this time.
- The LF/HF ratio decreases significantly only after stimulation of ST36 based on intensification of the blood pressure regulation, which is a related mechanism, as has been demonstrated in previous studies [4].
